# A regulatory similarity measure using the location information of transcription factor binding sites in *Saccharomyces cerevisiae*

**DOI:** 10.1186/1752-0509-8-S5-S9

**Published:** 2014-12-12

**Authors:** Wei-Sheng Wu, Ming-Liang Wei, Chia-Ming Yeh, Darby Tien-Hao Chang

**Affiliations:** 1 Department of Electrical Engineering, National Cheng Kung University, Tainan, 70101, Taiwan

## Abstract

**Background:**

Defining a measure for regulatory similarity (RS) of two genes is an important step toward identifying co-regulated genes. To date, transcription factor binding sites (TFBSs) have been widely used to measure the RS of two genes because transcription factors (TFs) binding to TFBSs in promoters is the most crucial and well understood step in gene regulation. However, existing TFBS-based RS measures consider the relation of a TFBS to a gene as a Boolean (either 'presence' or 'absence') without utilizing the information of TFBS locations in promoters.

**Results:**

Functional TFBSs of many TFs in yeast are known to have a strong positional preference to occur in a small region in the promoters. This biological knowledge prompts us to develop a novel RS measure that exploits the TFBS location information. The performances of different RS measures are evaluated by the fraction of gene pairs that are co-regulated (validated by literature evidence) by at least one common TF under different RS scores. The experimental results show that the proposed RS measure is the best co-regulation indicator among the six compared RS measures. In addition, the co-regulated genes identified by the proposed RS measure are also shown to be able to benefit three co-regulation-based applications: detecting gene co-function, gene co-expression and protein-protein interactions.

**Conclusions:**

The proposed RS measure provides a good indicator for gene co-regulation. Besides, its good performance reveals the importance of the location information in TFBS-based RS measures.

## Background

Identification of co-regulated genes are helpful for solving many biological problems such as unraveling the underlying molecular mechanisms of specific cellular functions, identifying functionally related proteins and dissecting the gene regulatory networks [1-3]. The first step toward identifying co-regulated genes is to define the regulatory similarity (i.e., the degree of co-regulation) of two genes. Gene regulation is a complex process, which involves various mechanisms: transcription factors (TFs) binding, miRNAs binding, epigenetic modifications, etc. Nowadays, various data related to these mechanisms, such as TF binding sites, miRNA binding sites and histone modification patterns, are available for gene regulation study. Among them, TF binding sites (TFBSs) have been the most widely used data. This is because that TFs binding to TFBSs in promoters is the most crucial and well understood step in gene regulation.

To date, many studies have been proposed to use TFBS data to measure the regulatory similarity (RS) of two genes [4-8]. However, existing TFBS-based RS measures consider the relation of a TFBS to a gene as a Boolean (either 'presence' or 'absence') without utilizing the information of TFBS locations. In yeast and human, functional TFBSs of many TFs are known to have a strong positional preference to occur in a small region in the promoters [9,10]. This biological knowledge prompts us to develop a novel RS measure that exploits the TFBS location information. Following Allocco et al.'s approach [11], the performances of different RS measures are evaluated by the fraction of gene pairs that are co-regulated (validated by the literature evidence deposited in the YEASTRACT database [12]) by at least one common TF under different RS scores. The experimental results show that the proposed RS measure was the best co-regulation indicator among the six compared RS measures. In addition, the co-regulated genes identified by the proposed RS measure are also shown to be able to benefit three co-regulation-based applications: detecting gene co-function, gene co-expression and protein-protein interactions.

## Methods

This study proposes a novel RS measure using the TFBS location information. This section first describes the datasets used in this study and five existing TFBS-based RS measures followed by the proposed RS measure.

### Datasets

Following previous studies in the literature, the promoter of a yeast gene in this study is defined as the intergenic region between this gene and its nearest non-overlapped upstream gene [13-18]. The genomic locations of the start and stop codons of 6604 genes of *Saccharomyces cerevisiae *(the budding yeast) were retrieved from Nagalakshmi et al.'s work [19]. The genomic locations of 422576 TFBSs of 163 yeast TFs were collected from the SwissRegulon database [20], which deposited high-quality TFBS datasets predicted using Bayesian probabilistic analysis. Users can choose different posterior probability cutoffs to control the quality of the retrieved TFBSs. This study adopted a moderate cutoff of 0.5 and included a section to discuss the influence of the TFBS quality to the proposed RS measure.

### Existing TFBS-based RS measures

Table 1 lists five existing TFBS-based RS measures of two genes, *a *and *b*. The first three RS measures do not consider the copies of TFBSs (namely a TF having multiple TFBSs is identical to that having one TFBS), while the last two do. In the context, TFs whose TFBSs exist in the promoter of *a *and *b *are denoted as *TF_a _*and *TF_b_*, respectively. TFs that have TFBSs in the promoters of both *a *and *b*, (*i.e*. *TF_a_*∩*TF_b_*) are named as common TFs. In the first group of RS measures, Garten et al. adopted the cumulative hypergeometric test to estimate the significance of the observed overlap between *TF_a _*and *TF_b _*in comparison with random expectation [4]. Veerla and Höglund adopted the Jaccard index to define the similarity of promoter organization between two genes [5]. This index calculates the RS as the size ratio of the intersection to the union of *TF_a _*and *TF_b_*. Shalgi et al. proposed a variant of Eq. (2) by replacing the denominator with the smaller size of *TF_a _*and *TF_b _*[6]. In the second group of RS measures, Park et al. used the proportion of TFBSs in common as the RS of two genes and introduced a penalty term for TFBSs appearing in only one gene's promoter [7]. Van Helden adopted the Poisson distribution to define the RS of two gene as the difference of the similarity score (1-the p-value of the observed TFBSs in common) and the dissimilarity score (the difference between the p-values of the observed TFBSs in *a *and in *b*) [8].

**Table 1:** Five existing TFBS-based RS measures

RS measure	Equation
Garten et al.^1^	$-\text{log}\left(\overset{\text{min}\left(m,n\right)}{\underset{x\geq k}{\sum }}\frac{\left(\begin{array}{c} m\\ x \end{array}\right)\left(\begin{array}{c} N-m\\ n-x \end{array}\right)}{\left(\begin{array}{c} N\\ n \end{array}\right)}\right)$ Eq. (1)
Veerla and Höglund	$\frac{\left|TF_{a}\cap TF_{b}\right|}{\left|TF_{a}\cup TF_{b}\right|}$ Eq. (2)
Shalgi et al.	$\frac{\left|TF_{a}\cap TF_{b}\right|}{\text{min}\left(\left|TF_{a}\right|,\left|TF_{b}\right|\right)}$ Eq. (3)
Park et al.^2^	$\begin{array}{cc} S & =\overset{2}{\underset{j=1}{\sum }}\gamma_{j}\underset{i}{\sum }{\left(f_{i}^{j}\right)}^{-1/2}\\ & \left[\left(\frac{2}{N_{1i}^{j}+N_{2i}^{j}}+\alpha \right)C_{i}^{j}-\beta \left(N_{1i}^{j}+N_{2i}^{j}\right)I_{\left\{C_{i}^{j}=0\right\}}\right] \end{array}$ Eq. (4)
van Helden^2^	$M^{ab}=S^{ab}-\alpha D^{ab}+\beta$ Eq. (5)

RS measures of two genes, *a *and *b*. *TF_a _*and *TF_b _*represent the TFs whose TFBSs exist in the promoter of *a *and *b*, respectively. ^1^In Eq. (1), *N *is the number of TFs whose binding sites are in the collected TFBS data, *m*=|*TF_a_*|, *n*=|*TF_b_*| and *k*=|*TF_a_*∩*TF_b_*|. ^2^Equations (4) and (5) only show the final equations of the two works. The equation details can be found in the original manuscripts [7,8].

### The proposed RS measure

**Figure 1:**
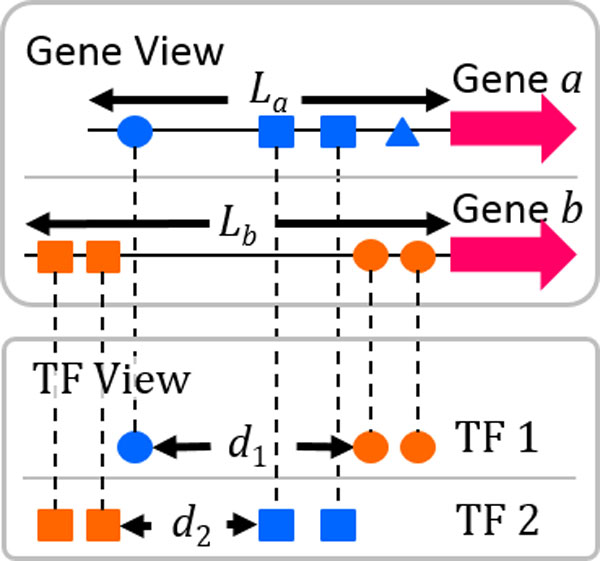
**Calculation details of the proposed RS measure**. TFBSs have different shapes for different TFs and have different colors for different genes where they locate. Gene View aligns the genes. TF View focuses on the TFBS locations associated to one common TF at a time. In this sample, there are three TFs (circle, square and triangle) and two (circle and square) of them are common TFs of the two genes. The longer promoter length *L *is *L_b_*. According to Eq. (6), the RS of the two genes is $\frac{1}{3}\left(\frac{L_{b}-d_{1}}{L_{b}}+\frac{L_{b}-d_{2}}{L_{b}}\right)$.

Equations (1)-(5) consider the relation of a TFBS to a gene as a Boolean (either 'presence' or 'absence') without utilizing the information of TFBS locations in the promoters. The biological knowledge that the biological relevance of TFBSs is highly related to their locations in the promoters [9,10] motivates us to introduce the TFBS location information into the RS measure as follows:

$\frac{1}{\left|TF_{a}\cup TF_{b}\right|}\underset{i\in TF_{a}\cap TF_{b}}{\sum }\frac{L-d_{i}}{L}$, Eq. (6)

where *L *is the longer promoter length of genes *a *and *b, i *is the *i*-th common TF that has TFBSs in the promoters of both *a *and *b*, and *d_i _*is the smallest distance between any two *i*-th common TF's TFBSs in different promoters. In this context, *d_i _*is called TFBS offset distance. A schematic explanation of Eq. (6) is shown in Figure 1, where TFBSs have different shapes for different TFs and have different colors for different genes where they locate. The two promoters of *a *and *b *are aligned by the start codons (Gene View). To compute *d_i_*, only the TFBSs of the *i*-th common TF are used and those of other TFs are ignored (TF View). In Figure 1, a small *d_i_*, which leads to a high RS, indicates that the TFBSs of the *i*-th common TF in the two promoters are in a similar region.

## Results and discussion

### Small TFBS offset distances imply high regulatory similarity

This study is motivated by the biological knowledge that functional TFBSs of many TFs in yeast are known to have a strong positional preference in the promoters [9]. Because the critical regions in the promoters that make TFBSs functional are unknown, Eq. (6) is actually based on a derived hypothesis: if the offset distance of two TFBSs of a common TF in two genes' promoters is small, the two TFBSs are prone to co-present in the critical regions and therefore be co-functional. To investigate the practicability of the above hypothesis, a relation analysis of the co-functionality and the TFBS offset distance was conducted as follows. As shown in Figure 1, a TFBS offset distance can be computed given a TF *t *and two genes *a *and *b*, denoted as a <*t, a, b*> tuple. In this analysis, the co-functionality related to a TFBS offset distance was defined as the ratio of tuples in which the literature evidences collected by the YEASTRACT database [12] showed that TF *t *regulates both *a *and *b *to all tuples. The detailed steps are listed below:

- For a TF *t*, all gene pairs <*a, b*> whose promoters have the TFBS of *t *were collected.

- The TFBS offset distance (as *d_i _*in Figure 1) of *t *relative to <*a, b*> was calculated.

- A tuple <*t, a, b*> was stored in the bucket of the TFBS offset distance, *B_d_*, where *d *is the TFBS offset distance of <*t, a, b*>.

- After repeating 1-3 for all TFs, each bucket contains all tuples having the same TFBS offset distance.

- Finally, the relation of *d *and the ratio of tuples in the bucket *B_d _*in which the literature evidences showed that TF *t *regulates both *a *and *b *to all tuples was plotted.

**Figure 2:**
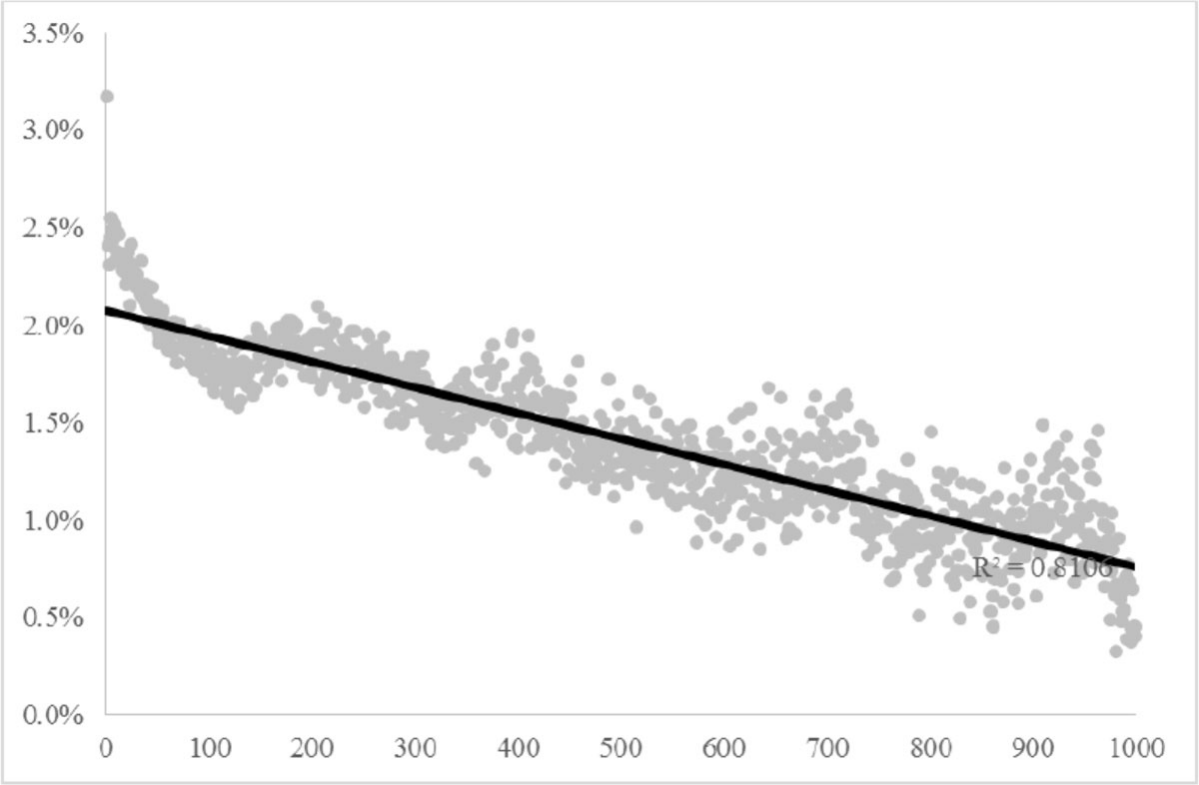
**TFBS offset distance vs. co-regulation tendency.** This figure shows the TFBS offset distance (*x*-axis, the *d_i _*in Figure 1) vs. the co-regulation tendency (*y*-axis).

The results are shown in Figure 2, where each point is a bucket, the *x*-axis is the TFBS offset distance, while *y*-axis is the ratio of tuples in which the literature evidences showed that TF *t *regulates both *a *and *b *to all tuples. Figure 2 shows an obvious linear relation (R^2 ^= 0.8106), which suggest that the above hypothesis is practically usable. Reviewing Eq. (6), it implements this concept by incorporating *d_i_*, where a common TF which has a smaller TFBS offset distance (*d_i_*) has a larger value of $\frac{L-d_{i}}{L}$.

### The proposed RS measure is a good co-regulation indicator

**Figure 3:**
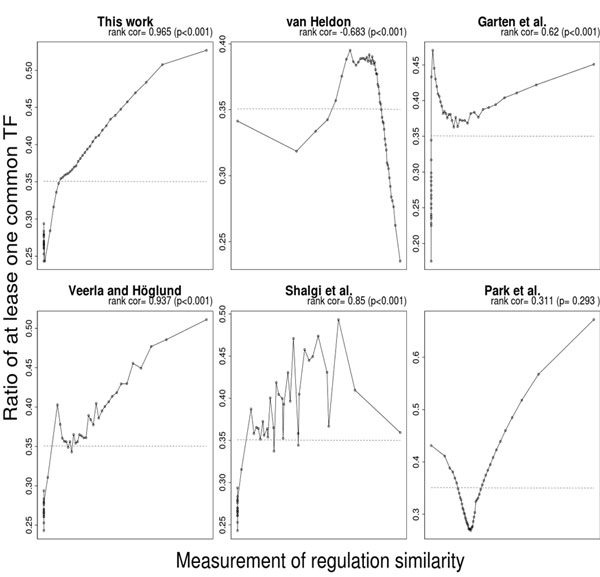
**Comparison of six regulatory similarity measures**. This figure is plotted as follows. First, a subset of one million samples was randomly selected from the evaluation dataset of 19495447 gene pairs. Second, the scores of the selected gene pairs are calculated. Third, the correlation plot of one subset is generated. Each point represents 2% of gene pairs (each figure contains 50 points) in that subset. In a correlation plot, *x *and *y *are the average RS score and the fraction of gene pairs that are co-regulated (validated by the literature evidence deposited in the YEASTRACT database) by at least one common TF, respectively. The gene pairs were sorted by the RS score. For example, the rightest point represents the 2% gene pairs of the highest RS scores. Finally, the three steps are repeated 100 times and this figure shows an average plot of 100 correlation plots. The dashed line indicates a random predictor in which RS scores are randomly assigned.

Following Allocco et al.'s approach [11], this study evaluated TFBS based RS measures by the fraction of gene pairs that are co-regulated (validated by the literature evidence) by at least one common TF under different RS scores. From the 6604 yeast genes retrieved from Nagalakshmi et al.'s work [19], 359 genes having no TFBSs were excluded. The remaining 6245 genes formed 19496890 gene pairs, where 1443 head-to-head gene pairs (both genes in such a pair share the same promoter) were further excluded. Finally, the remaining 19495447 gene pairs were used as the evaluation dataset. Figure 3 shows the results of Eqs. (1-6) on the evaluation dataset. In Figure 3, the *x*-axis is the RS score obtained by different RS measures and the *y*-axis is the fraction of gene pairs that are co-regulated (validated by the literature evidence) by at least one common TF to all gene pairs under the corresponding RS scores.

The results show that the proposed RS score is highly correlated to the likelihood of a gene pair to be co-regulated by at least one common TF. The plot of the proposed RS measure (Figure 3a) is increasing and smooth at most regions except the few points at left. It achieved a significantly higher R^2 ^(0.963) of Spearman rank correlation than random expectation with p-value less than 0.001. In comparison with other RS measures, the R^2 ^of the proposed measure is significantly higher than those of other existing RS measures (see Table 2). Since the unique feature of the proposed RS measure is introducing TFBS location information, this shows that TFBS location information is useful in calculating regulatory similarity between two genes. The previous section showed the underlying hypothesis as well as a numerical evidence. The results in this section, furthermore, show that the implementation of Eq. (6) of the hypothesis works. Although the implementation of Eq. (6) may incorrectly increase the weights of TFBSs co-present in the non-critical regions, it effectively decreases the weights of those present in the critical region of one gene but in a non-critical region of the other gene.

**Table 2:** Significance of performance difference of the proposed RS measure against five methods

RS measure	P-value
van Helden	5.36 × 10^-244^
Veerla and Höglund	3.23 × 10^-83^
Garten et. al.	4.82 × 10^-213^
Park et. al.	4.88 × 10^-231^
Shalgi et. al.	8.04 × 10^-137^

P-values are calculated by one-tailed t-test.

### The effects of TFBS qualities

The SwissRegulon database [20], of which the TFBS data were used in this study, provides users a parameter of posterior probability to control the quality of the obtained TFBSs. Actually most resources of TFBS locations provide parameters such as ChIP-chip p-value and phylogenetic conservation and let users to choose the most appropriate values for their applications [13,17,21]. This section aims to figure out whether the TFBS quality affects the performance of the proposed RS measure and, if it does affect, what TFBS qualities are suggested.

**Figure 4:**
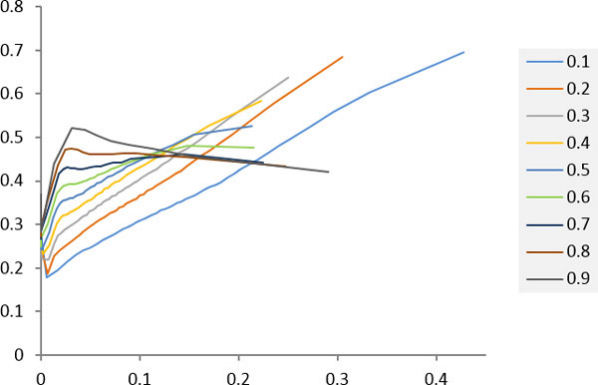
**Effects of different TFBS qualities on the proposed RS measure**. Each point represents 2% of gene pairs (each figure contains 50 points) of which *x *and *y *are the average RS score and the ratio of gene pairs that are co-regulated (validated by the literature evidence deposited in the YEASTRACT database) by at least one common TF, respectively. The gene pairs were sorted by the RS score. Different lines represent the results using SwissRegulon TFBS data of the corresponding posterior probability cutoffs.

Figure 4 shows the results of the proposed RS measures using different SwissRegulon posterior probability cutoffs. The obvious turn at the region of 0.00~0.05 of the curves corresponding to high cutoffs (0.8 and 0.9) reveals that the proposed RS measure (*x*-axis) were badly correlated to the likelihood of a gene pair to be co-regulated by at least one common TF (*y*-axis). The curves of the next two lower cutoffs (0.7 and 0.6) were smoother but still had a small peak around *x *= 0.15. As the cutoff dropped, the correlation of the *x*-axis and *y*-axis was getting stronger. These results suggest a strange conclusion: the proposed RS measure requires TFBS quality worse than a threshold. This conclusion could be explained by the TFBS quantity (Table 3). It is reasonable that the quality cutoff also affected the quantity. The TFBS quantity of cutoff 0.1 was about three times to that of cutoff 0.7 and ten times to that of cutoff 0.9. The results suggest that, instead of TFBS quality, the proposed RS measure was more sensitive to the drastic change of TFBS quantity. With enough TFBS quantity, the proposed RS measure is robust to current TFBS data, even using the one with the lowest quality (cutoff 0.1).

**Table 3:** TFBS qualities and quantities

**Quality**^1^	Quantity^2^	#Genes^3^	Density^4^
0.1	313746	6332	49.5
0.2	220938	6311	35.0
0.3	169405	6292	26.9
0.4	134111	6272	21.4
0.5	106299	6245	17.0
0.6	84066	6192	13.6
0.7	65011	6080	10.7
0.8	47955	5903	8.1
0.9	30785	5527	5.6

^1^Posterior probability cutoff in the SwissRegulon database. The higher cutoff, the better TFBS quality. ^2^Number of annotated TFBS locations in the SwissRegulon database under the corresponding cutoff. ^3^Number of genes whose promoter has at least one annotated TFBS in the SwissRegulon database under the corresponding cutoff. ^4^Quantity / #Genes.

### Case study

This section uses a case (yeast *CCT8*) to explain the performance advantage of the proposed RS measure. *CCT8 *is a subunit of the cytosolic chaperonin Cct ring complex. In this case study, yeast *CCT8 *was of interest and its co-regulated genes were wanted. For this purpose, the RSs of all yeast genes to *CCT8 *were calculated and the 30 highest ranked genes were considered as co-regulated gene candidates of *CCT8 *(Table 4). To dig in the uniqueness of the proposed RS measure, we focused on a candidate, *RPN8*, which is only identified by the proposed RS measure but not identified by the other five compared RS measures. We further dug into which genes were ranked before *RPN8 *(therefore pushed it out the candidate list) by the other RS measures and found an interesting opponent gene, *RSC1*, against *RPN8*.

**Table 4:** Co-regulated genes of *CCT8 *identified by the proposed RS measure

Gene list	Uniqueness^1^
*RPN8, THI12, GTF1, GBP2, NOP7*, YOR262W*, NUP84, MDM32, TMA108, NUP85, URB2, MSO1*	0
*THR4, PRE8, SEC65, ISN1*	1
*RCF1, MRPL16, TIF11, RPN3, CYM1*, YGL010W*, URA7, RPA12*, YNL144W-A*, SCL1, EMC4*	2
*CSH1*, YLR030W*, RPL15A*	3

^1^Number of RS measures among the five compared ones (Table 1) that also identified the genes. For example, the uniqueness of *RPN8 *is 0, indicating that it is only identified by the proposed RS measure but not identified by the five compared ones.

**Figure 5:**
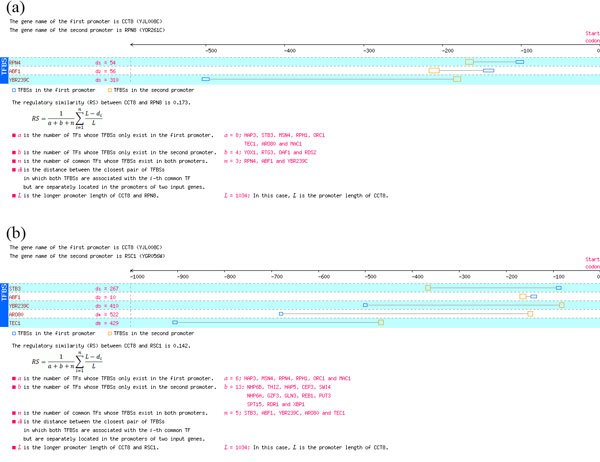
Case study (a) The aligned promoters of *CCT8 *and *RPN8*. (b) The aligned promoter of *CCT8 *and *RSC1*.

Table 5 shows the rank orders of the two genes (*RPN8 *and *RSC1*) among all yeast genes by the similarity to *CCT8 *using different RS measures. In this table, the proposed RS measures gave a better rank of *RPN8 *(#29) than that of *RSC1 *(#117), but all the other five RS measures gave a reverse rank order. To further investigate the details, the promoters of *CCT8, RPN8 *and *RSC1 *were plotted (Figure 5). Figure 5a depicts the aligned promoters of *CCT8 *and *RPN8*; while Figure 5b depicts the aligned promoter of *CCT8 *and *RSC1*. The number of common TFs of *CCT8 *and *RPN8 *is three, and the number of common TFs of *CCT8 *and *RSC1 *is five. This is why the other TFBS-based RS measures give a better rank of *RSC1 *than that of *RPN8*. However, two of the three common TFs of *CCT8 *and *RPN8 *has small TFBS offset distance (Rpn4 and Abf1) and only one of the five common TFs of *CCT8 *and *RPN8 *has small TFBS offset distance (Abf1). Since the proposed RS measure is the only one that considers the information of TFBS locations, this is why the proposed RS measure gave a different rank order of *RPN8 *and *RSC1 *to the other measures.

**Table 5:** Ranks of *RPN8 *and *RSC1 *against *CCT8*

RS measure	*RPN8* ^1^	Order^2^	*RSC1* ^3^
This work	29	<	117
van Helden	3162	>	244
Veerla and Höglund	37	>	31
Garten et. al.	61	>	33
Park et. al.	126	>	26
Shalgi et. al.	402	>	330

^1^Rank of the RS score of *RPN8 *against *CCT8 *among the RS scores of all yeast genes against *CCT8*. ^2^Symbol '<' indicates that the RS score of *RPN8 *against *CCT8 *is higher than that of *RSC1*; symbol '>' indicates that the RS score of *RPN8 *against *CCT8 *is lower than that of *RSC1*. ^3^Rank of the RS score of *RSC1 *against *CCT8 *among the RS scores of all yeast genes against *CCT8*.

To justify the correctness of the rank order, the biological relevance of common TFs were analyzed. In this study, a TF is defined biologically relevant to a gene if the literature evidences obtained from the YEASTRACT database show that the TF regulates the gene. In Figure 5, all TFs with small TFBS offset distances are biologically relevant to both target genes (Rpn4 and Abf1 to both *CCT8 *and *RPN8 *in (a) and Abf1 to both *CCT8 *and *RSC1 *in (b)). Furthermore, all the other TFs, which have large TFBS offset distances, are not simultaneously relevant to both downstream genes. This suggests the correctness of the proposed RS measure as well as the importance of incorporating the information of TFBS locations.

### Good RS measure benefits co-regulation-based applications

Co-regulated genes are considered to influence many biological behaviors and co-regulation measures have been used in various applications [22,23]. The section "The proposed RS measure is a good co-regulation indicator" shows that the proposed RS is a good co-regulation index over the five competitors. This section discusses whether this leads to a better result in three co-regulation-based applications: detecting gene co-function, gene co-expression and protein-protein interactions.

In this study, the scenario of detecting gene co-function, gene co-expression and protein-protein interactions using gene co-regulation was designed as follows. First, users have a target gene of interest. The RS score of the target gene against each gene in the genome is calculated. The *n *genes with the highest RSs are called the regulatory neighborhood (RN) to the target gene and *n *is called the neighborhood size. Then the degree of co-function of the RN is evaluated using the functional enrichment score proposed by Reimand et al. [24], denoted as FES in this study. In FES, genes are considered to perform similar biological functions if they have similar Gene Ontology (GO) terms [25]. The degree of co-expression of the RN is evaluated by the co-expression score proposed by Yang and Wu [26], denoted as CES in this study. CES is the average of the pairwise expression correlations in the RN. The degree of protein-protein interactions of the RN is evaluated by the interaction enrichment score proposed by Reimand et al. [24], denoted as IES in this study. IES measures the tendency of forming protein complex modules of a RN.

**Figure 6:**
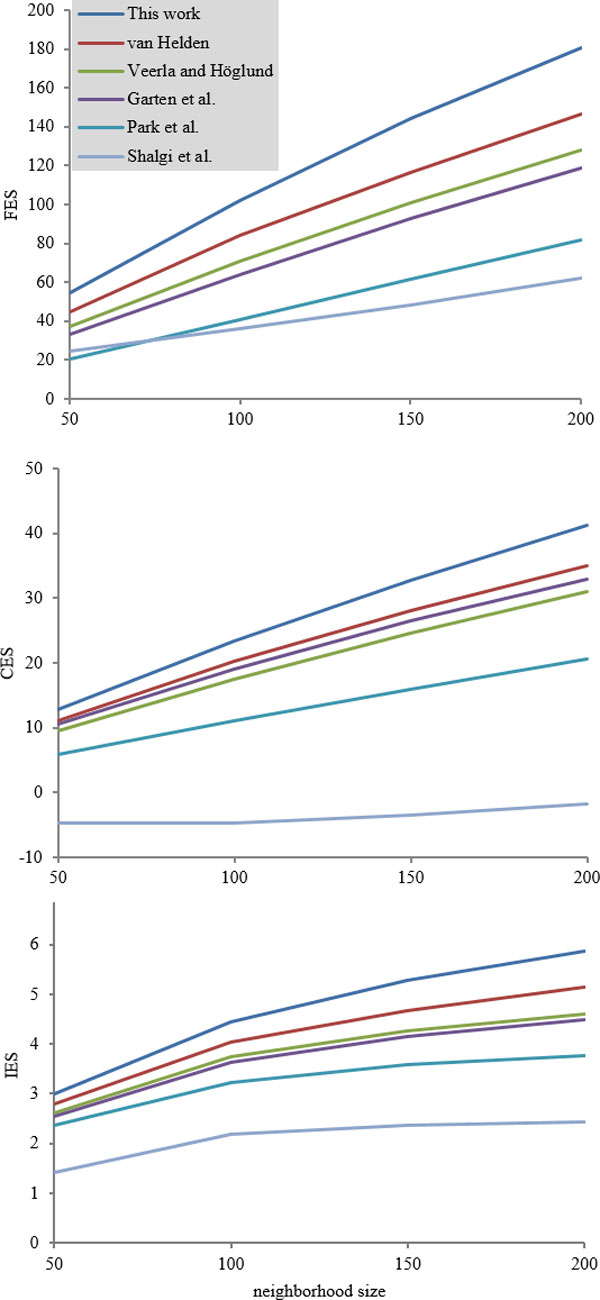
**Comparison of six regulatory similarities on three applications**. The *x*-axis is the neighborhood size of the regulatory neighborhood (RN) while the *y*-axis is the degree of (top) gene co-function calculated using the functional enrichment score (FES), (middle) gene co-expression calculated using the co-expression score (CES) and (bottom) protein-protein interactions using the interaction enrichment score (IES) of the RN identified by the RS measure.

The results of the proposed RS measure and the five existing RS measures in the three applications are shown in Figure 6 and Table 6. The proposed RS measure achieved the highest performance among all the compared RS measures in all applications and all neighborhood sizes. In all three applications, the RS measures of van Helden, Veerla and Höglund and Garten et al. had similar performance and were the second best group.

**Table 6:** Comparison of six regulatory similarities on three applications

RS measure	FES^1^	CES^2^	**IES**^3^	Average^4^
This work	**1**	**1**	**1**	**1.0**
van Helden	2	2	2	2.0
Veerla and Höglund	3	4	3	3.3
Garten et al.	4	3	4	3.7
Park et al.	5	5	5	5.0
Shalgi et al.	6	6	6	6.0

^1^The RS measures are ranked in terms of gene co-function (the area under curve (AUC) of Figure 6a). ^2^The RS measures are ranked in terms of gene co-expression (the AUC of Figure 6b). ^3^The RS measures are ranked in terms of protein-protein interaction (the AUC of Figure 6c). ^4^The average of the ranks by gene co-function, gene co-expression and protein-protein interactions.

## Conclusions

This study proposed a novel measure that can compute the regulatory similarity (RS) of two genes using the location information of transcription factor binding sites. Based on the documented regulation associations between TFs and genes in the YEASTRACT database, this study has shown that the proposed RS measure is a good co-regulation indicator. Furthermore, its good performance can benefit to three co-regulation-based applications. The proposed RS measure will be helpful for unraveling the underlying molecular mechanisms of specific cellular functions and dissecting the gene regulatory networks.

## Authors' contributions

WSW and DTHC conceived the research topic, provided essential guidance, developed the algorithm and wrote the manuscript. MLW and CMY performed all the simulations. All authors read and approved the final manuscript.

## Declaration

The publication charges of this article were funded by Ministry of Science and Technology of Taiwan grant NSC 102-2221-E-006-085-MY2.

This article has been published as part of *BMC systems Biology *Volume 8 Supplement 5, 2014: Proceedings of the 25th International Conference on Genome Informatics (GIW/ISCB-Asia): Systems Biology. The full contents of the supplement are available online at http://www.biomedcentral.com/bmcsystbiol/supplements/8/S5.
